# K13 Propeller Mutations in Plasmodium falciparum Populations in Regions of Malaria Endemicity in Vietnam from 2009 to 2016

**DOI:** 10.1128/AAC.01578-16

**Published:** 2017-03-24

**Authors:** Nguyen Thuy-Nhien, Nguyen Kim Tuyen, Nguyen Thanh Tong, Nguyen Tuong Vy, Ngo Viet Thanh, Huynh Thuy Van, Pham Huong-Thu, Huynh Hong Quang, Maciej F. Boni, Christiane Dolecek, Jeremy Farrar, Guy E. Thwaites, Olivo Miotto, Nicholas J. White, Tran Tinh Hien

**Affiliations:** a Oxford University Clinical Research Unit, Wellcome Trust Major Overseas Programme, Ho Chi Minh City, Vietnam; b Institute of Malariology, Parasitology, and Entomology, Quy Nhon, Vietnam; c Center for Tropical Medicine and Global Health, Nuffield Department of Medicine, University of Oxford, Oxford, United Kingdom; d Mahidol Oxford Tropical Medicine Research Unit, Mahidol University, Bangkok, Thailand; e Centre for Genomics and Global Health, Wellcome Trust Centre for Human Genetics, Oxford, United Kingdom; f Wellcome Trust Sanger Institute, Hinxton, United Kingdom

**Keywords:** K13 mutation, artemisinin resistance, frequency, Plasmodium falciparum, RSA, parasite clearance, Vietnam

## Abstract

The spread of artemisinin-resistant Plasmodium falciparum compromises the therapeutic efficacy of artemisinin combination therapies (ACTs) and is considered the greatest threat to current global initiatives to control and eliminate malaria. This is particularly relevant in Vietnam, where dihydroartemisinin-piperaquine (DP) is the recommended ACT for P. falciparum infection. The propeller domain gene of K13, a molecular marker of artemisinin resistance, was successfully sequenced in 1,060 P. falciparum isolates collected at 3 malaria hot spots in Vietnam between 2009 and 2016. Eight K13 propeller mutations (Thr474Ile, Tyr493His, Arg539Thr, Ile543Thr, Pro553Leu, Val568Gly, Pro574Leu, and Cys580Tyr), including several that have been validated to be artemisinin resistance markers, were found. The prevalences of K13 mutations were 29% (222/767), 6% (11/188), and 43% (45/105) in the Binh Phuoc, Ninh Thuan, and Gia Lai Provinces of Vietnam, respectively. Cys580Tyr became the dominant genotype in recent years, with 79.1% (34/43) of isolates in Binh Phuoc Province and 63% (17/27) of isolates in Gia Lai Province carrying this mutation. K13 mutations were associated with reduced ring-stage susceptibility to dihydroartemisinin (DHA) *in vitro* and prolonged parasite clearance *in vivo*. An analysis of haplotypes flanking K13 suggested the presence of multiple strains with the Cys580Tyr mutation rather than a single strain expanding across the three sites.

## INTRODUCTION

Plasmodium falciparum resistance to artemisinin has emerged in six countries of the Greater Mekong subregion: Cambodia (1, 2), Thailand (3), Myanmar, the Lao People's Democratic Republic, China (4), and Vietnam (5). In many areas along the Cambodia-Thailand border, P. falciparum has become resistant to most available antimalarial drugs. In Vietnam, where the number of clinical malaria cases reported declined from 1,672,000 with 4,650 deaths in 1991 to 19,252 with 3 deaths in 2015 (6), the artemisinin combination therapy (ACT) dihydroartemisinin-piperaquine (DP) has been the first-line treatment of falciparum malaria since 2005. The success of the national malaria control program led to the initiation of Malaria Elimination by the Year 2025, a project endorsed by strong political commitment to scale up malaria control activities. However, the emergence of artemisinin resistance now threatens the achievements of the malaria control program and poses a great challenge to the malaria elimination aspirations of Vietnam.

To support and develop an effective strategy to cope with drug resistance before it spreads to unaffected areas where malaria is not currently endemic, it is necessary to characterize its emergence and spread. Artemisinin resistance is associated with nonsynonymous single-nucleotide polymorphisms (SNPs) in the beta-propeller domain of a kelch protein (known as K13), encoded by the gene PF3D7_1343700 on chromosome 13 of the P. falciparum genome (4, 7). Several different K13 point mutations have been identified in parasites across the Greater Mekong area (5). It has also been shown that in this geographical area K13 mutations most frequently emerge in parasites possessing a genetic background characterized by a number of mutations in other genes, notably, *arps10* V127M, *fd* D193Y, *mdr2* T484I, and *crt* N326S (8).

A 5-year-monitoring study with both *in vivo* and *in vitro* testing was started in 2010 on the basis of the recommendations of the Global Plan for Artemisinin Resistance Containment (9). Here we report on the identification of K13 mutations and their prevalence in the P. falciparum populations in three southern provinces of Vietnam, Binh Phuoc, Ninh Thuan, and Gia Lai, over the period from 2009 to 2016 and their association with changes in the efficacy of ACTs in Vietnam.

## RESULTS

A total of 1,060 samples from patients with P. falciparum malaria, confirmed by laboratory microscopy, were collected from June 2009 to January 2016 in the three provinces. Of these, 767 (72%) were from Binh Phuoc Province, 188 (18%) were from Ninh Thuan Province, and 105 (10%) were from Gia Lai Province (see Fig. S1 in the supplemental material). The propeller domain of the P. falciparum K13 gene was successfully sequenced from all 1,060 samples. K13 propeller domain mutations were found in 26% (278/1,060) of the samples and affected 8 different amino acid positions (Thr474Ile, Tyr493His, Arg539Thr, Ile543Thr, Pro553Leu, Val568Gly, Pro574Leu, and Cys580Tyr) (Table S1). There was no evidence that any parasite carried more than one K13 mutation.

### Prevalence of K13 mutations in P. falciparum parasites in Binh Phuoc Province from 2009 to 2016.

**FIG 1:**
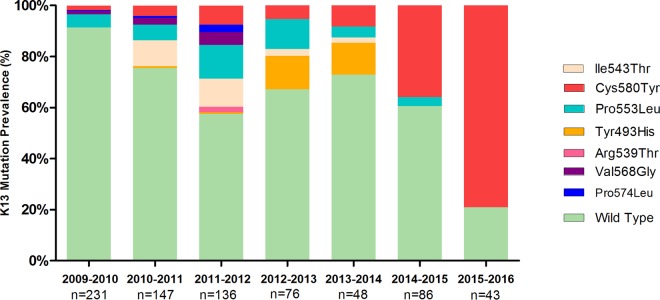
Prevalence of K13 mutations in P. falciparum parasites in Binh Phuoc Province, 2009 to 2016.

A set of 767 samples was collected from clinical trials and observational studies from different study sites in Binh Phuoc Province (Dong Xoai Hospital, Phuoc Long Hospital, and the Bu Gia Map and Dak O Health Centers). K13 propeller mutations were observed in 29% (222/767) of the samples collected between 2009 and 2016 and covered eight amino acid positions (Thr474Ile, Tyr493His, Arg539Thr, Ile543Thr, Pro553Leu, Val568Gly, Pro574Leu, and Cys580Tyr) (Fig. 1). The proportion of infections with strains with K13 mutant genotypes increased from 8.7% (20/231) in 2009 to 79.1% (34/43) in 2016 (*P* < 0.001).

The changes in the frequencies of strains with K13 mutations in Binh Phuoc Province from 2009 to 2016 are shown in Fig. S2. The prevalence of Cys580Tyr mutants increased from 1.7% (4/231) in the 2009-2010 season to 79.1% (34/43) in the 2015-2016 season (*P* < 0.001). No other K13 mutants showed significant increases in frequency during this period. In contrast to the rise in the prevalence of the Cys580Tyr mutant, the Ile543Thr mutant decreased in frequency from 10.2% (15/147) in 2010-2011 to 2.1% (1/48) in 2013-2014 and subsequently disappeared. Similarly, the prevalence of strains with the Tyr493His mutation rose from 0.7% (1/147) during the period from 2010 to 2012 to 12.5% (6/48) in 2013-2014, but the allele was not detected thereafter. The Pro553Leu mutant also experienced an initial rise in frequency, from 5.2% (12/231) in 2009-2010 to 15.4% (21/136) in 2011-2012, but it has become less common since then. The remaining mutations were detected only at low frequencies (<5%): Val568Gly and Pro574Leu between 2009 and 2012 and Arg539Thr in 2011-2012.

### Prevalence of P. falciparum strains with K13 mutations in Ninh Thuan and Gia Lai Provinces.

**FIG 2:**
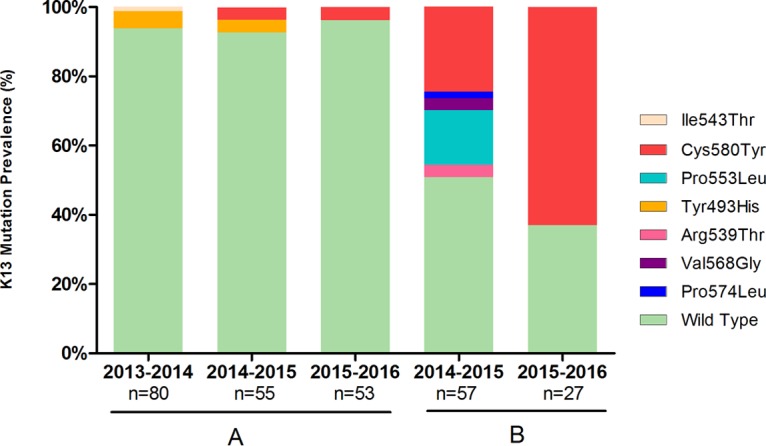
Prevalence of K13 mutations in P. falciparum parasites in Ninh Thuan (A) and Gia Lai (B) Provinces. Results for only two seasons in Krong Pa, Gia Lai, are shown. Samples from the first season were from a different commune (Iato, Gia Lai).

A set of 188 samples was collected from the Phuoc Thang District in Ninh Thuan Province between 2013 and 2016. K13 mutations were found in only 11/188 (6%) isolates, and these comprised mutations at three different alleles (Tyr493His, Ile543Thr, and Cys580Tyr). At this site, mutants with the Tyr493His mutation appeared to have been replaced by mutants with the Cys580Tyr mutation over the three sampling seasons. However, there appeared to be no significant rise in the frequency of these mutations over the same time period (Fig. 2A).

In Gia Lai Province, 105 samples were collected during three malaria seasons: in Iato Commune during the 2011-2012 season and in Krong Pa Hospital between 2014 and 2016. No K13 mutation was found in the 21 samples collected during the 2011-2012 season. In contrast, a high prevalence of K13 mutants was found during the 2014-2015 season, with 24.6% of samples (14/57) carrying the Cys580Tyr mutation, 15.8% (9/57) of samples carrying the Pro553Leu mutation, and 8.8% (5/57) of samples containing lower-frequency mutations, including Arg539Thr, Val568Gly, and Pro574Leu. In the 2015-2016 season, the Cys580Tyr mutation was the only K13 mutation detected and was found in 63% of the samples collected (17/27). The overall prevalence of K13 mutations in Gia Lai was 43% (45/105 samples) (Fig. 2B).

### Correlation between K13 mutations and parasite clearance *t*_1/2_.

**FIG 3:**
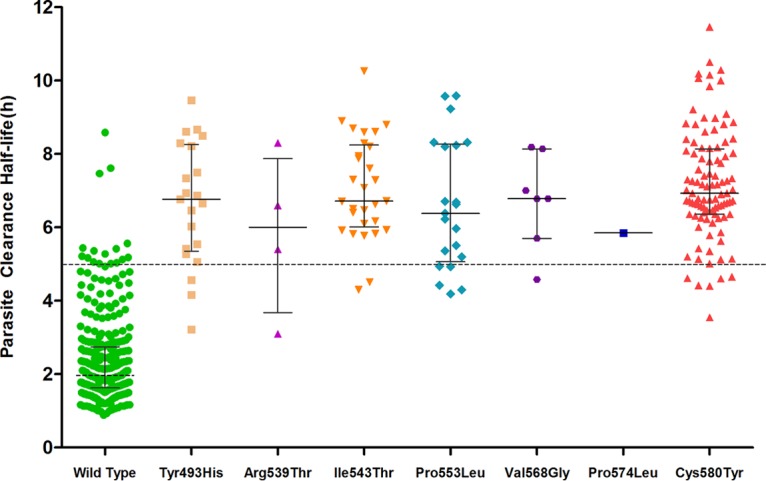
Association between K13 mutations and parasite clearance half-life in Vietnam, 2009 to 2016. The median (interquartile range) half-life for each mutation (shown as gray lines) is as follows: 1.96 h (1.63 to 2.73 h) for the wild type, 6.8 h (3.69 to 7.42 h) for Tyr493His, 6 h (4.81 to 7.04 h) for Arg539Thr, 6.7 h (5.9 to 8.02 h) for Ile543Thr, 6.4 h (4.39 to 7.08 h) for Pro553Leu, 6.8 h (6.22 to 7.59 h) for Val568Gly, and 6.9 h (6.33 to 8.14 h) for Cys580Tyr.

The association between K13 mutations and the estimated clinical parasite clearance half-life (*t*_1/2_) is shown in Fig. 3. Almost all patients infected with mutants with mutated K13 alleles had a *t*_1/2_ of >5 h. The half-lives of wild-type parasites (parasites without any K13 propeller mutations) were shorter than those of parasites carrying K13 mutations (*P* < 0.001).

### Correlation between K13 mutation and *ex vivo* DHA exposure.

**FIG 4:**
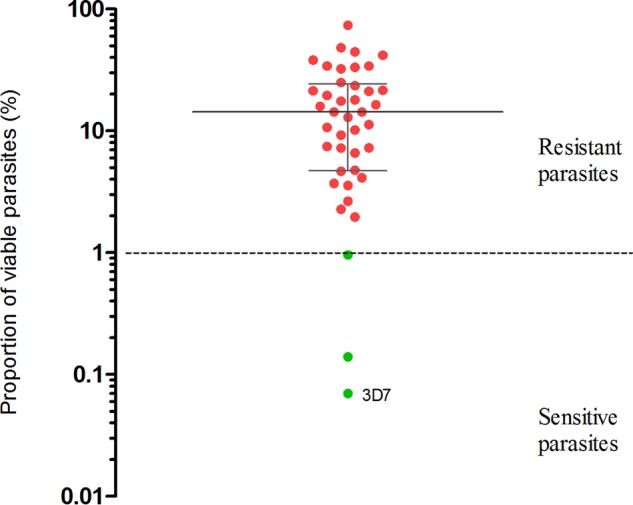
Ring-stage survival after exposure to dihydroartemisinin. Forty parasites were tested. Red dots, parasites with the Cys580Tyr mutation; green dots, parasites without the Cys580Tyr mutation. The median and interquartile range of the proportion of viable parasite are shown as gray lines.

Among the 40 parasite isolates tested by the ring-stage survival assay (RSA) (10), 2 (5%) were found to be sensitive to DHA and 38 (95%) were resistant. All parasites with survival rates of over 1% carried the Cy580Tyr mutation, while sensitive parasites were found to be the K13 wild type. The association between the K13 mutation and the parasite survival rate is shown in Fig. 4.

### Haplotype diversity of Cys580Tyr mutants in Binh Phuoc, Ninh Thuan, and Gia Lai Provinces.

**FIG 5:**
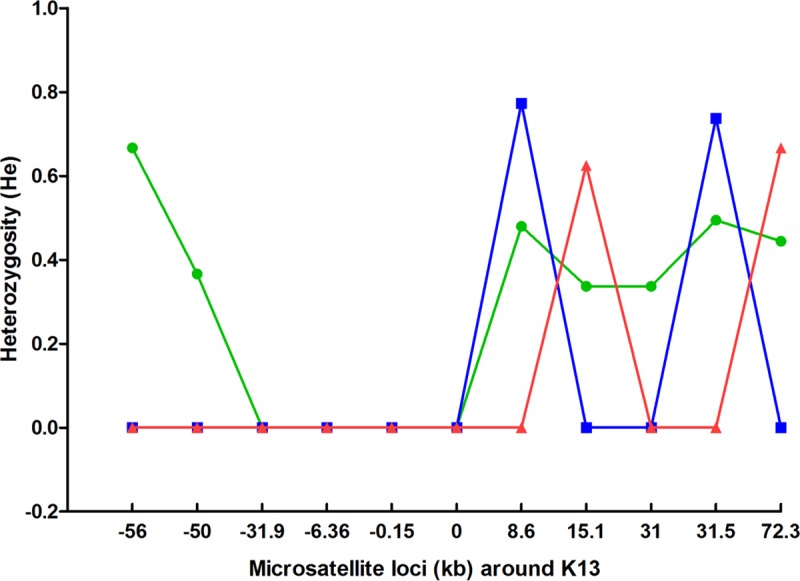
Allelic heterozygosity at microsatellite loci flanking the K13 gene in Cys580Tyr mutant parasites from Binh Phuoc (green), Ninh Thuan (red), and Gia Lai (blue) Provinces.

Twelve microsatellite loci in the regions flanking the K13 propeller gene of the Cys580Tyr mutant parasites were assayed (Table S2). No results could be obtained from two of the assayed loci (−3.74 kb and 3.4 kb). For each geographical site, the allelic heterozygosity (*H_e_*) at each of the remaining 10 loci was calculated. The extremely low heterozygosity in the upstream region flanking K13 suggests that, at each of the three sites, Cys580Tyr mutants have common origins (Fig. 5). However, the haplotypes seen in Binh Phuoc Province were distinct from those seen in the other two provinces, suggesting multiple origins of the Cys580Tyr mutation. In Binh Phuoc, we noted the presence of two distinct common haplotypes with identical core alleles, suggesting either independent origins of the Cys580Tyr mutations in genetically related parasites or recent recombination events proximal to K13 in mutants with a single origin. In Ninh Thuan and Gia Lai, we observed very similar core haplotypes flanking K13, suggesting that mutants with mutations at these two sites may have a common origin. However, at these two sites, we also observed considerable allelic diversity in the downstream flanking regions, which may indicate an earlier origin of the Cys580Tyr mutation in these samples than in those from Binh Phuoc.

## DISCUSSION

Since the reduced susceptibility of P. falciparum to artemisinin was first observed in western Cambodia (1), it has emerged in multiple parts of the Greater Mekong subregion, posing a major threat to regional elimination efforts. Numerous independent K13 propeller mutations associated with resistance have emerged independently across the region (10, 11), and the frequencies of some mutations, notably, Cys580Tyr, have risen in multiple geographical areas. The current study reports on the prevalence and the frequency of K13 mutations in three regions of southern Vietnam where malaria is endemic, determined using samples collected between 2009 and 2016. We detected point mutations at eight locations (Thr474Ile, Tyr493His, Arg539Thr, Ile543Thr, Pro553Leu, Val568Gly, Pro574Leu, and Cys580Tyr) which have been previously reported in Asia (12).

This cohort study in Vietnam shows that the proportion of isolates of P. falciparum with the K13 mutation increased rapidly from 8.6% (20/231) in the 2009-2010 season to 79.1% (34/43) in the 2015-2016 season. The incidence of malaria did not significantly decrease from 2009 to 2015 in the three areas where samples were collected (6), suggesting that the increase in the population with K13 mutations was genuine. The high proportion of isolates with K13 mutations might thus be interpreted to be a result of a decrease in the number of artemisinin-sensitive cases of P. falciparum malaria among the total number of cases of malaria in those regions. The clinical impact of this genetic change has been high: the appropriate clinical and parasitological response (APCR) rates for DP treatment were 93% and 86% in 2010 (13) and 2014, respectively, but the rate dropped to 78% in 2015 (14). The results of the present study confirm the spread of the phenotype with a long clearance half-life as well as an increase in the frequency of various K13 genotypes.

One particularly interesting feature was the increase in the predominance of the Cys580Tyr mutation at all sites, a pattern similar to that observed in neighboring Cambodia (8), Laos (15), and Thailand (16). One key question is whether K13 mutant parasites—and Cys580Tyr mutants in particular—were imported from other countries in the region or whether they emerged independently in Vietnam as a result of local drug pressure. A study which included several parasites from Binh Phuoc Province included in the present study (8) revealed that Vietnamese samples carrying the Tyr493His, Ile543Thr, and Cys580Tyr mutations had long K13 flanking haplotypes identical to those in Cambodian parasites carrying the same mutations. This suggests that K13 mutations in parasites from both sides of the Vietnam-Cambodia border share an ancestry, although it could not be definitively determined in which direction these strains have spread. In contrast, the same analysis showed that Pro553Leu mutant parasites may have originated independently in Binh Phuoc. Besides, K13 mutations often arise on a genetic background with mutations in genes including *arps10* V127M, *fd* D193Y, *mdr2* T484I, and *crt* N326S (8). Many of the sequenced samples from Binh Phuoc Province were included in the earlier analysis (8), which concluded that K13 mutations in Binh Phuoc Province emerge exclusively only in parasites that carry this background.

Previous analyses also showed that during the period from 2012 to 2013, Tyr493His mutant parasites in Binh Phuoc established a founder population similar to those observed in Western Cambodia, but the levels of genetic similarity between clones were higher, which was likely the result of extreme inbreeding (8). The present study shows that, in spite of the very elevated clearance *t*_1/2_ values exhibited by its members, the Tyr493His founder population could no longer be detected and appeared to have been superseded by a population with the Cys580Tyr mutation. One speculative hypothesis for this changeover is that mutations such as Tyr493His may be potent in affecting the clearance *t*_1/2_ value but cause a loss of fitness that can be tolerated only in the presence of a combination of compensatory changes which can be maintained in the population only by extreme inbreeding. On the other hand, it is possible that the Cys580Tyr mutation requires fewer compensatory changes and, therefore, that Cys580Tyr mutants might be impacted to a lesser extent by recombination with other strains. Greater flexibility in mixing with local populations could explain why a very rapid rise in the frequency of the Cys580Tyr mutation was also observed in Gia Lai, where the prevalence of this allele reached 63% in only 2 years, raising worrying prospects for future failures of ACTs in this area. Encouragingly, the proportion of parasites with K13 mutations in Ninh Thuan Province has remained very low, although the two mutations observed (Tyr493His and Cys580Tyr) are the same as those that dominate elsewhere. One hypothesis for the difference between the proportion of parasites with K13 mutations in Ninh Thuan Province and the other two provinces is the frequency of travel across the shared border with Cambodia. The populations in Ninh Thuan travel only to forests which are located within the province, while people from Binh Phuoc and Gia Lai usually cross the border with Cambodia. Local minority populations rarely travel to work in other provinces. Our analysis of the haplotypes flanking the K13 gene suggests that there are multiple origins of the Cys580Tyr mutation circulating at the three sites. Interestingly, parasites in Ninh Thuan Province appear to carry haplotypes more similar to those of parasites in Gia Lai Province than to those of parasites in Binh Phuoc Province. In addition, parasites at these sites exhibit greater haplotypic diversity, while Binh Phuoc Cys580Tyr mutants can be split into two groups with very similar haplotypes. One hypothesis that could explain the observed patterns is that the Cys580Tyr strain of one haplotype now dominant in Binh Phuoc may have recently been introduced there and may not yet have reached Gia Lai and Ninh Thuan, where the Cys580Tyr mutants of the other haplotype had earlier origins. It is possible that, in Binh Phuoc, these earlier mutants have been replaced by the more competitive recent Cys580Tyr strain, similar to the findings for the previously mentioned Tyr493His founder population. If this hypothesis is correct, it is likely that the haplotypes seen in Cys580Tyr mutants in Binh Phuoc will spread further in the future. Continued genetic surveillance of the parasite populations over the next few years will be crucial to further our understanding of the gene flow associated with antimalarial resistance.

An association between K13 mutations and the clinical parasite clearance half-life was also observed in this study. A longer half-life was correlated with the presence of the Tyr493His, Ile543Thr, Pro553Leu, and Cys580Tyr mutations, in agreement with the findings described in a WHO report and other studies (7, 17). In addition, *ex vivo* testing using the ring-stage survival assay (RSA) provided confirmatory evidence of artemisinin resistance in Vietnamese K13 mutant parasites. Taken together, these data support the use of two laboratory markers, those obtained by analysis of K13 and RSA, to confirm artemisinin resistance in P. falciparum in Vietnam.

One early contention about the use of day 3 positivity and the parasite clearance half-life as markers of artemisinin resistance was that different human populations had variable levels of immunity to malaria and that a slow clearance rate in a naive population could simply be an indication of a lower level immunity compared with that in comparator populations. Of the sites where parasite clearance half-life measurements have been taken so far, Vietnam is likely to have one of the lowest levels of population immunity. The incidence of malaria has declined significantly in Vietnam over the past 25 years (18, 19). In Binh Phuoc Province, the annual incidence declined from 2.11 cases per 1,000 population in 2013 to approximately 1.68 cases per 1,000 population in 2014. With less than 0.5% of the population experiencing a clinical case of malaria in a year, it is likely that the vast majority of the population is immunologically naive to this disease. However, results presented here show that K13 mutant parasites in Vietnam may become resistant independently of the human host that they infect. In the future, however, it may be interesting to compare the parasite clearance half-lives of parasites carrying the same K13 allele in different parts of Vietnam and its neighboring countries.

Future elimination and control strategies must be planned in the Greater Mekong region in the context of rapidly spreading K13 mutations. Genetic surveillance can detect rising frequencies of these mutations, which would be a clear indicator that ACT treatments are under selective pressure. A further major issue is that longer parasite clearance times will cause an increased number of parasites to be exposed to the ACT partner drugs and increase the chances that resistance to partner drugs will emerge. This is currently a major concern for Vietnam, because a high rate of DP failures has been reported from multiple sites in Cambodia (20, 21), and *ex vivo* analyses suggest that these are caused by parasites resistant to piperaquine (22). Clearly, the independent emergence of piperaquine resistance or its spread from other countries would threaten the strategies of Vietnam's national malaria control program. It is imperative to identify molecular markers for piperaquine resistance, so that the parasite population can be monitored and the national malaria control program can be supported with timely information to allow it to adjust its strategies. Furthermore, it will be important to survey the parasite populations for markers of resistance to other potential partner drugs, so that alternative ACTs can be evaluated. Vietnam has now moved into the pre-malaria elimination phase of malaria control as a result of the deployment of ACTs and the success of its national program. The continued success of this program and the future elimination of malaria from the country will depend on sustaining the efficacy of ACTs and, therefore, on carefully monitoring the spread of drug resistance markers.

## MATERIALS AND METHODS

### Sample collection.

**FIG 6:**
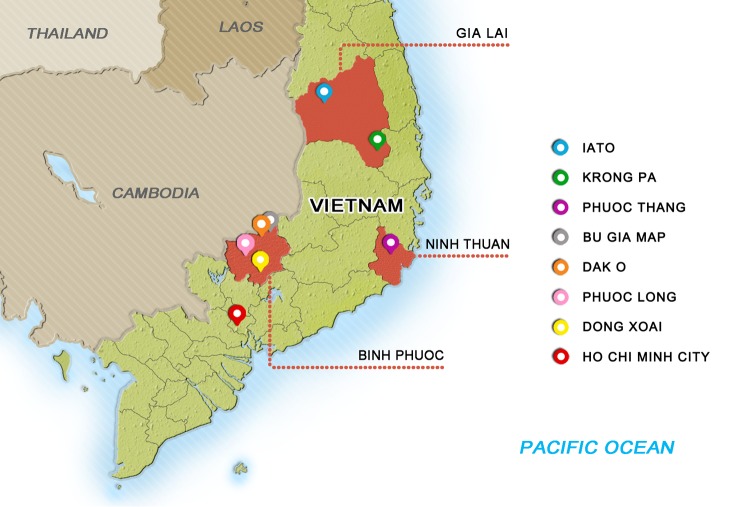
Locations of the three regions in Vietnam where malaria is endemic. The regions of endemicity (Binh Phuoc, Ninh Thuan, and Gia Lai Provinces) are shown in red. The locations of sample collection are marked.

K13 mutations were identified in dried blood spots or whole-blood samples taken from consenting patients with uncomplicated falciparum malaria who participated in clinical trials (4, 13, 14) and observational studies conducted between 2009 to 2016 in Binh Phuoc, Ninh Thuan, and Gia Lai Provinces in Vietnam. Briefly, blood was collected on admission from patients with levels of parasitemia ranging from 1,000 to 200,000 parasites/μl. Patients were treated with DP according to national treatment guidelines, as described in the study protocol (5, 13, 14). Blood smears were prepared and checked every 6 h until there were two consecutive negative readings. The study sites are shown in Fig. 6. Samples were grouped by the malaria season in which they were collected (from June to May of the following year) rather than by calendar year. The study protocol, informed consent documents, relevant supporting information, and all patient recruitment information were approved by the Ethics Committees of the Ho Chi Minh and Qui Nhon Institutes of Malaria, Parasitology and Entomology and the Oxford University Tropical Research Ethics Committee.

### K13 gene amplification and sequencing.

The total genomic DNA was extracted from the parasite isolates by use of an automated extraction system (a MagNA Pure 96 instrument) and a MagNA Pure 96 DNA and viral NA small-volume kit (Roche, Switzerland).

A subset of whole-blood samples (*n* = 238) was depleted of leukocytes through CF11 filtration (23) prior to DNA extraction to reduce human DNA contamination. DNA from these samples was submitted to the Sanger Institute, Hinxton, United Kingdom, for whole-genome sequencing on an Illumina HiSeq platform. Following alignment of the sequencing reads for these samples against the sequence of 3D7 V3 reference genome (ftp://ftp.sanger.ac.uk/pub/pathogens/Plasmodium/falciparum/3D7/3D7.latest_version/version3/), the K13 alleles were derived from read counts at a nonsynonymous SNP in the K13 propeller domain, using a procedure described previously (8).

The remaining samples (*n* = 822) were genotyped by capillary sequencing of the K13 propeller domain. First, nested PCR was used to amplify the K13 gene using the Platinum PCR supermix (Life Technologies, Ontario, Canada) in 25-μl reaction mixtures containing 22.5 μl of reaction buffer, 10 mM (each) primer, and 1.7 μl of template DNA. The PCR thermocycling conditions of the first round were 2 min at 95°C, 30 s at 95°C, and then 30 cycles of 30 s at 95°C, 1 min at 55°C, and 90 s at 72°C and a final extension of 4 min at 72°C. The second round of PCR differed in the amplification step by the use of 35 cycles of 30 s at 95°C, 30 s at 55°C, and 1 min at 72°C. The primer set used was described in previous reports (6, 25). The PCR products were then purified by use of a QIAquick PCR purification kit (Carlsbad, CA) and finally sequenced using an Applied Biosystems 3130 XL 16 capillary sequencer. The K13 sequences were assembled with the ContigExpress program and aligned with the K13 gene sequence of the 3D7 clone (PF3D7_1343700) using the MEGA (version 5.0) program to identify any SNPs present.

### Microsatellite loci genotyping.

Samples with the Cys580Tyr mutation collected in 2015 and 2016 were used for microsatellite genotyping. Twelve microsatellite loci around the K13 gene (PF3D7_1343700; downstream, 3.4 kb, 8.6 kb, 15.1 kb, 31.0 kb, 31.5 kb, and 72.3 kb; upstream, −0.15 kb, −3.7 kb, −6.36 kb, −31.9 kb, −50.0 kb, and −56.0 kb) were genotyped by the protocol of Talundzic et al. (24). The sizes of the amplification products were determined by capillary electrophoresis on an Applied Biosystems 3130 XL sequencer (Applied Biosystems). To determine genetic diversity, the expected heterozygosity (*H_e_*) was estimated on the basis of the findings for all microsatellite loci around K13 using the Excel Microsatellite tool kit (version 3.1.1) (11).

### RSA.

The *ex vivo* responses to DHA in 40/43 parasite samples (collected from malaria patients involved in the DP efficacy monitoring study in 2015 and 2016 in Binh Phuoc Province) were measured by a ring-stage survival assay (RSA) (10). The results were evaluated by calculation of the survival rate of parasites after 6 h of drug exposure.

### Data analysis.

Comparison of the frequencies of K13 mutations in each season was performed by the chi-square test. Parasite clearance half-life data were collected from relevant clinical trials conducted by OUCRU-VN, as reported elsewhere (4, 13, 14), and calculated using the WWARN parasite clearance estimator (https://www.wwarn.org/pce). All analyses were performed with the statistical software Stata (version 7.0) and Prism (version 5.0; GraphPad, USA).

## Supplementary Material

Supplemental material
