# Supplementary material for: K13 Propeller Mutations in Plasmodium falciparum Populations in Regions of Malaria Endemicity in Vietnam from 2009 to 2016
Source: Antimicrob Agents Chemother. 2017 Mar 24;61(4):e01578-16. doi: 10.1128/AAC.01578-16 (PMC5365681; doi:10.1128/AAC.01578-16)
Supplement: Supplemental material [file supp_61_4_e01578-16__index.html]

Supplemental material 

# K13 Propeller Mutations in Plasmodium falciparum Populations in Regions of Malaria Endemicity in Vietnam from 2009 to 2016

## Supplemental material

- Supplemental file 1 -

  Supplemental Tables S1 and S2 and Figures S1 and S2

  PDF, 386K
