# Supplementary material for: K13 Propeller Mutations in Plasmodium falciparum Populations in Regions of Malaria Endemicity in Vietnam from 2009 to 2016
Source: Antimicrob Agents Chemother. 2017 Mar 24;61(4):e01578-16. doi: 10.1128/AAC.01578-16 (PMC5365681; doi:10.1128/AAC.01578-16)
Supplement: Supplemental material [file AAC.01578-16_zac004176047s1.pdf]

Supplement Materials for AAC01578-16R2 "**K13-Propeller Mutations in *Plasmodium falciparum* Populations in Malaria Endemic Regions of Vietnam from 2009 to 2016**"

Table S1. Number of samples with K13-propeller mutations in *P.falciparum* populations in Vietnam 2009-2016.

**Mutations**	**No**	**Binh Phuoc**							**Ninh Thuan**			**Gia Lai**	
		2009 - 2010	2010 - 2011	2011 - 2012	2012 - 2013	2013 - 2014	2014 - 2015	2015 - 2016	2013 - 2014	2014 - 2015	2015 - 2016	2014 - 2015	2015 - 2016
Thr474Ile	1	0	1	0	0	0	0	0	0	0	0	0	0
Tyr493His	24	0	1	1	10	6	0	0	4	2	0	0	0
Arg539Thr	5	0	0	3	0	0	0	0	0	0	0	2	0
Ile543Thr	34	0	15	15	2	1	0	0	1	0	0	0	0
Pro553Leu	65	12	9	21	9	2	3	0	0	0	0	9	0
Val568Gly	16	3	4	7	0	0	0	0	0	0	0	2	0
Pro574Leu	5	1	1	2	0	0	0	0	0	0	0	1	0
Cys580Tyr	128	4	6	10	4	4	31	34	0	2	2	14	17

Table S2: Alleles at K13 flanking microsatellite loci of Cys580Tyr mutant parasites from study sites in Vietnam. NT: Ninh Thuan province (n=4); BP: Binh Phuoc province (n=30); GL: Gia Lai province (n=18). To improve pattern visualization, at any one locus each allele is assigned a different background colour. Blank cells indicate that the assay did not produce a result.

	**Microsatellite loci**									
**Sample**	**72.3**	**31.5**	**31**	**15.1**	**8.6**	**-0.15**	**-6.36**	**-31.9**	**-50**	**-56**
**NT01**	205	194	304	145	276	195	284	209	258	
**NT02**	174	194	304	117	276	195	284		258	
**NT03**	179	194	304	140	276	195	284	209	258	
**NT04**		194	304	140	276		284	209	258	
**BP01**	178	194	304	137		197				
**BP02**	178	194	304	137		197	286	209	261	
**BP03**		194	304	137		197	286	209	261	
**BP04**	173	194	304	137		197		209	261	
**BP05**		194	304	137				209	261	
**BP06**		194	304	137		197	286	209	261	
**BP07**		194	304	137		197		209	261	
**BP08**		194	304	137		197		209	261	
**BP09**		194	304	137	278	197		209	261	
**BP10**		194	304	137			286	209	261	
**BP11**		194		137		197	286	209	261	
**BP12**		194	304	137		197		209	261	
**BP13**		194	304	137		197		209	261	
**BP14**		194	304	137		197		209	261	
**BP15**		192	304	137	278	197	286	209	261	
**BP16**		192	304	137		197	286	209	261	
**BP17**		192	304	137	278	197	286	209	261	
**BP18**		192	304	137	278	197	286	209	261	
**BP19**		192	304		278	197	286	209	261	
**BP20**		192	304	137	278	197	286	209	261	
**BP21**		192	304	137	278	197	286	209	261	163
**BP22**		192	304	137	278	197	286	209	261	164
**BP23**		192	304	137	278	197	286	209	261	181
**BP24**		194	306	141	288	197	286	209	258	
**BP25**		192	306	141	288	197	286	209	258	
**BP26**		192	306	141	288	197	286	209	258	
**BP27**		192	306	141	288	197	286	209	258	
**BP28**		192	306	141	288	197	286	209	258	

**BP29**		194	306	141	288	197	286	209	258	
**BP30**									258	
**GL01**			304	150		195	284	209	261	
**GL02**		186	304	150	278		284		261	
**GL03**		202	304	150	278	195	284	209	261	
**GL04**		194	304	150	278	195	284	209	261	
**GL05**		194	304	150	278	195	284	209	261	
**GL06**		189	304	150	274	195	284	209	261	
**GL07**		189	304	150	274	195	284	209	261	
**GL08**		189	304	150		195	284	209	261	
**GL09**		189	304	150	274	195	284	209	261	
**GL10**		194	304	150	285	195	284		261	
**GL11**		194	304	150	288	195	284	209	261	
**GL12**		194	304	150	285	195	284		261	
**GL13**		198	304	150	288	195	284	209	261	
**GL14**		198	304	150	276	195	284	209	261	
**GL15**		198	304	150	276	195	284	209	261	
**GL16**		198	304	150	288	195	284	209	261	
**GL17**			304	150	288	195	284	209	261	
**GL18**			304	150	288	195	284	209	261	

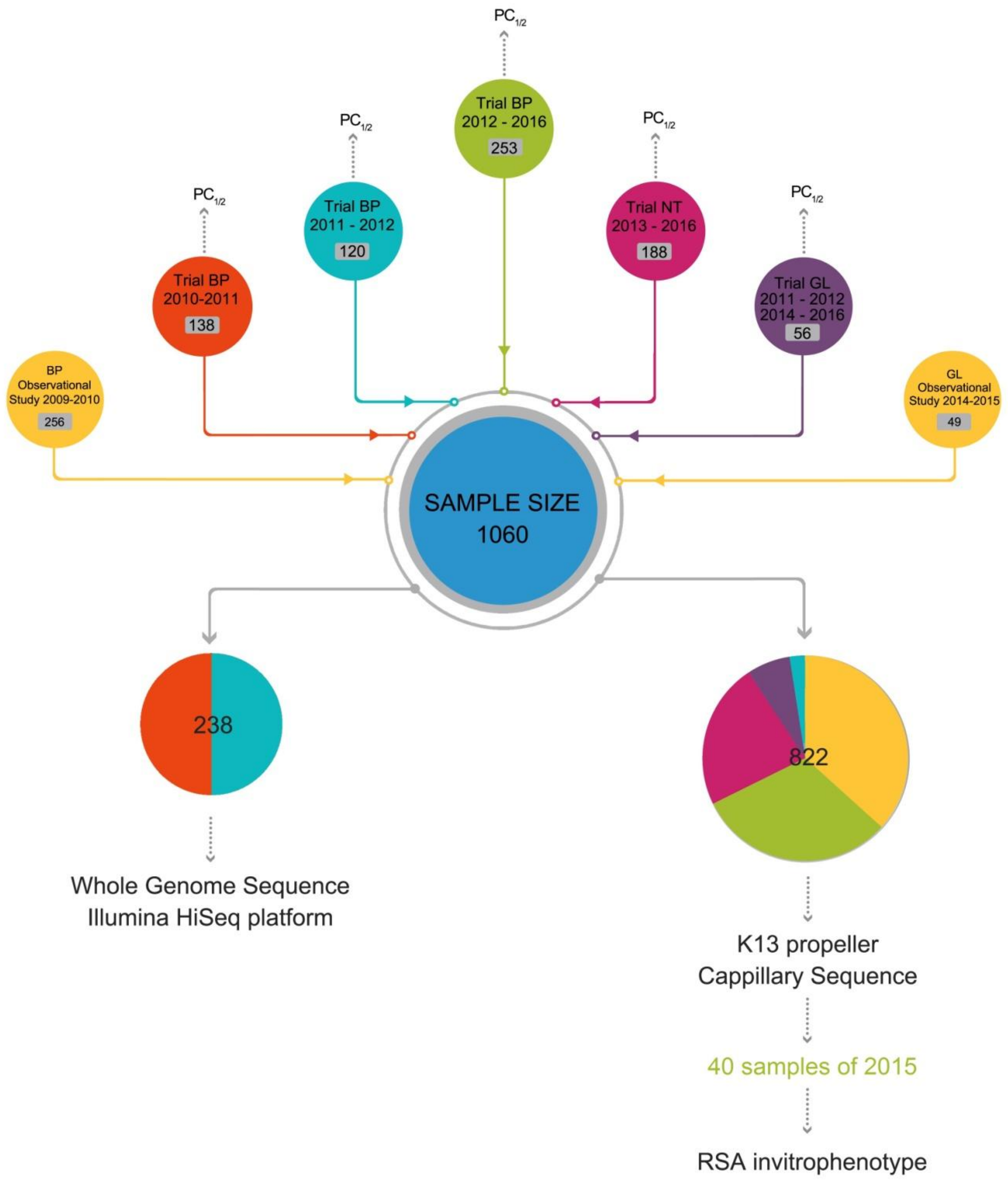

Figure S1: Flow diagram of sample processing

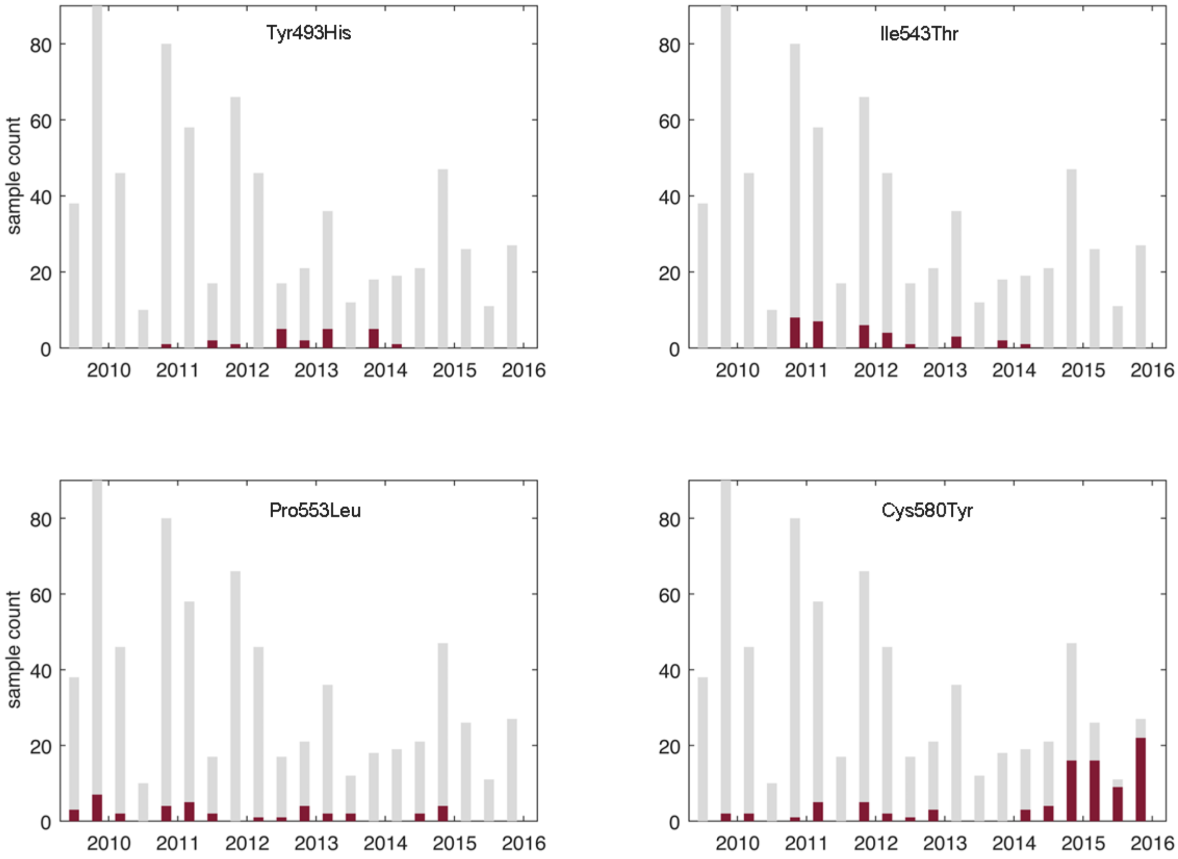

Figure S2. Percentage of four K13 polymorphisms in Binh Phuoc province 2009-2016. Sample counts disaggregated to 4-month periods. The gray bar shows the total number of samples collected during the given period, and the red portion of the gray bar shows the number with a particular mutation. The tick marks are placed on January 1 of each year. In the period Sep-Dec 2009, 146 samples were collected.
